# Photobiomodulation Therapy in the Treatment of Oral Mucositis—A Case Report

**DOI:** 10.3390/medicina58050618

**Published:** 2022-04-29

**Authors:** Przemysław Jabłoński, Mikołaj Musiał, Rafał Wiench, Natalia Stefanik, Cyprian Olchowy, Jacek Matys, Dariusz Skaba, Kinga Grzech-Leśniak

**Affiliations:** 1 Specialist Medical Centre Periomed Przemysław Jabłoński, 42-100 Klobuck, Poland; periomedklobuck@interia.pl; 2 Department of Orthodontics, Faculty of Medical Sciences in Zabrze, Medical University of Silesia, 40-055 Katowice, Poland; mikolajmusial1992@gmail.com; 3 Department of Periodontal Diseases and Oral Mucosa Diseases, Faculty of Medical Sciences in Zabrze, Medical University of Silesia, 40-055 Katowice, Poland; rwiench@sum.edu.pl (R.W.); nstefanik@sum.edu.pl (N.S.); dskaba@sum.edu.pl (D.S.); 4 Department of Oral Surgery, Medical University of Wroclaw, 50-425 Wroclaw, Poland; cyprian.olchowy@umed.wroc.pl (C.O.); jacek.matys@umed.wroc.pl (J.M.); 5 Department of Orthodontics, Technische Universitat Dresden, 01307 Dresden, Germany; 6 Department of Periodontics, School of Dentistry, Virginia Commonwealth University, VCU, Richmond, VA 23298, USA

**Keywords:** cancer complications, diode laser, supportive cancer care, side effects

## Abstract

In 2021, our group published a laboratory study on the impact of PBM on human gingival fibroblasts. The in vitro results confirmed the fact that the appropriately selected wavelength and properly selected parameters of the laser settings can increase cell proliferation, modulate inflammatory markers, and decrease the susceptibility of human gingival fibroblasts to apoptosis. Therefore, this case report was aimed at the clinical evaluation of the proposed settings and treatment regimen in a very difficult situation of an immunocompromised patient with extensive changes and stagnation of symptoms for many weeks. A 65-year-old man, during his oncological treatment, was diagnosed with oral mucositis grade 3 according to the World Health Organization and National Cancer Institute scales. Due to pain sensation, long-lasting and not healing oral lesions, and problems with solid food intake, he was qualified for laser photobiomodulation therapy. For the management of oral lesions, a diode laser 635 nm (Smart^M^Pro, Lasotronix, Poland) was intraorally applied at an energy density of 4 J/cm^2^, the 20 s of irradiation, the output power of 100 mW, and in continuous wave mode. Seven treatment procedures were performed two times a week using the spot technique in contact and non-contact mode. Within 21 days of monotherapy, all ailments disappeared. The patient was also able to reuse dental dentures and return to a solid diet. The obtained results confirm the efficiency of at least 3 PBM protocols. Our case shows that the use of PMB therapy contributes to faster healing of painful oral lesions in oncological patients, and thus the treatment time and return to the appropriate quality of life is shorter.

## 1. Introduction

Oral mucositis (OM) is one of the most common complications of oncological treatment using chemotherapy (CT) or radiotherapy (RT) or both simultaneously, especially for cancerous lesions located in the head and neck area. Changes in the oral mucosa can occur in up to 80% of patients undergoing high-dose chemotherapy assisted by hematopoietic stem cell transplantation (HSCT) [1]. The progression of complications depends on the type and location of the tumor, the type, dose, duration of the administered cytostatic drug, and the individual patient’s sensitivity [1,2].

There are many assessment scales for rating the severity of OM. The most commonly used have been proposed by the World Health Organization (WHO) and the National Cancer Institute (NCI) [3]. The WHO grades OM according to clinical observations and the effect of mucositis on the possibility of food intake. The NCI-CTCAE (Common Terminology Criteria for Adverse Events) version 4.03 scale relies on clinical observations, patients’ subjective symptoms, the ability to eat, and overall patient safety [3].

The principles of managing OM are focused on pain control and relief, oral cavity disinfection, alleviation of the mucosal dryness symptom, choosing the right therapy in the case of secondary infection, treatment of bleeding, and nutritional deficiencies [4,5]. Thus far, no universal procedure has been found to prevent the onset of inflammatory complications of the mucosa during oncological treatment. All the studies to date indicate the vital role of good oral hygiene in preventing this condition, and comprehensive education and dental control visits are essential in patients at risk of OM [5,6]. The advancement of the disease, the vastness of lesions, the complexity of the problem, and the lack of a golden remedy require searching for alternative or supportive methods of OM prevention and treatment. The following procedures can be considered: mucosal cooling, the use of human keratinocyte-1 growth factor (Palifermin) and keratinocytes-2 (Repifermin), or photobiomodulation (PBM) [5,6,7,8]. Therapy with the electromagnetic waves of laser light deserves special attention because successful laser application can count in many fields of dentistry [9,10,11,12,13,14].

A significant number of studies have proven the beneficial effect of PBM on reducing the adverse effect of cytostatics on the oral mucosa by reducing the inflammation process, reducing pain, preventing fibrosis, and enhancing wound healing and tissue regeneration [2,15,16,17,18,19,20]. In 2021, our group published a laboratory study on the impact of PBM on human gingival fibroblasts [21]; the in vitro results confirmed the fact that the appropriately selected wavelength and properly selected parameters of the laser settings can increase cell proliferation, modulate inflammatory markers, and decrease the susceptibility of human gingival fibroblasts to apoptosis through the downregulation of apoptosis-related genes. Moreover, the data indicate that the modulation of CD90 and CD105 mesenchymal markers expression can reflect the possible changes in the differentiation status of irradiated fibroblasts. Our observation is that it is also worth emphasizing that the above-described methods of PBM’s influence on fibroblasts are particularly visible, in terms of biochemical parameters, in a regular cycle of irradiation, and not in a single exposure. Only a series of at least three regularly conducted irradiations or more resulted in a long-term in vitro effect. Therefore, this case report was aimed at the clinical evaluation of the proposed settings and treatment regimen in a very difficult situation of an immunocompromised patient with extensive changes and stagnation of symptoms for many weeks.

## 2. Case Report

The study was conducted with the approval of the Local Bioethics Committee (Institutional Review Board affiliated with the Medical University of Silesia, Katowice, Poland; protocol resolution no. KNW/0022/KBI/70/1/18). A 65-year-old man was referred to the Department of Periodontal Diseases and Mucosa in Zabrze. The patient complained of severe pain, xerostomia, and the presence of inflammatory lesions in the oral cavity that had lasted for several months. Chronic ailments and moderate oral dryness complicated the possibility of speech, solid food intake, and wearing dentures. In March 2015, the patient was diagnosed with a diffuse large B-cell lymphoma (DLBCL), revised international prognostic index (R-IPI)-1, of the subclavicular lymph nodes on the right side, and qualified for eight cycles of RCHOP chemotherapy (Rituximab, Cyclophosphamide, Doxorubicin, Vincristine, Prednisolone). In November 2018, because of the active process of hyperplasia of lymph nodes on both sides of the diaphragm—which was diagnosed by PET (Positron Emission Tomography)—another six cycles of the same therapy were used. Due to the lack of remission, in May 2019, was decided to perform HSCT. A control PET in September 2019 showed the progression of the neoplastic disease. In December 2019, it was decided to continue chemotherapy according to the RB regimen (Bendamustine 100 mg, Rituximab 660 mg) for 4 cycles. Oral symptoms appeared during this medication. The patient’s accompanying chronic diseases were moderate hypertension and hypothyroidism, both pharmacologically stabilized.

There was no skin pathology in the extraoral examination. However, in both labial commissures, 20 by 20 mm ulcers were visible which covered a few millimeters of cutaneous surface and passed onto the labial and buccal mucosa, surrounded by erythema. Lymphoedema involved submental, submandibular, and superficial cervical nodes. The remaining lymph nodes of the head and neck area were imperceptible. There was no compression soreness of the supraorbital and infraorbital nerves and no trismus.

In the intraoral examination, extensive ulcerations with erythema were observed on the lateral surface of the tongue, descending to its abdominal surface on the left side (45 × 40 mm), also on the right palatal arch, descending into the retromolar triangle region and on the lateral surface of the pharynx (about 30 × 20 mm)—covered with a layer of fibrin. The dorsal surface of the tongue was covered with a thick white coating. All oral mucosa was dry, shineless, and sticky to the touch. Quantitation of resting and stimulated saliva flow showed, respectively, 0.2 and 0.4 mL/min. For pain sensation, a visual analog scale (VAS) was used where 0 represented no pain at all and 10 represented the most significant pain. The pain was the strongest during food intake (VAS = 7) and caused significant dietary modifications (mixed food) and prolonged mealtimes. The patient lost 4 kg of body weight over three months due to pain and dysphagia. According to the anamnesis and examination of the oral cavity, the patient was diagnosed with oral mucositis grade 3 according to the WHO scale and grade 3 according to the NCI scale.

The patient did not use any topical medications in the oral cavity, but systematically took Encorton 20 mg, IPP 40 mg, Cipropol 250 mg, Heviran 400 mg, and Kaldyum 600 mg. To exclude possible secondary infection, the patient was referred for bacteriological and mycological examination. An oncologist was asked for permission for PBM therapy.

The patient was first instructed to use an oral mouth rinse Fomukal (Vipharm, Poland) 6–10 times per day. On the second visit (after seven days), due to the lack of improvement in symptoms, after excluding bacterial and fungal infections and after obtaining permission for laser treatment, PBM therapy was one end only the treatment of choice. According to the detailed recommendations of MASCC/ISOO/ESMO (Multinational Association of Supportive Care in Cancer/International Society of Oral Oncology/European Society for Medical Oncology) regarding the prophylaxis and treatment of OM in patients undergoing radio and/or chemotherapy with HSCT according to the criteria ASCO (American Society of Clinical Oncology), the use of keratinocyte growth factors is recommended as prophylaxis 3 days before the start of therapy and/or 3 days after its completion. In our case, the patient was referred much later, which would significantly reduce the efficacy of such therapy. Additionally, mucosal cooling, recommended only for patients receiving high doses of melphalan, which our patient did not receive, was not implemented). A diode laser 635 nm (SmartPro, Lasotronix, Poland) was used with an energy density of 4 J/cm^2^, 20 s of irradiation, output power of 100 mW, and continuous wave mode average power density: of 199.04 mW/cm^2^. The procedure was performed using an 8 mm diameter glass tip with a flat surface, spot area: 0.5024 cm^2^. The procedure was performed using an 8 mm diameter glass tip with a flat surface. Due to the patient’s capabilities, a series of 2 times per week treatments were scheduled.

The spot technique (point-by-point) was used: intraoral contact mode (undamaged mucosa) and intraoral or transcutaneous contact/non-contact mode (damaged mucosa/dermis), and 3 mm was maintained between irradiation points. Before each session, the tip of the laser was disinfected with a solution of 70% alcohol. For the management of lesions, pain, and oral dryness, the following areas were irradiated: 4 points on the dorsal side of the tongue, 3 points on each lateral border of the tongue, 4 points on the inferior surface of the tongue, 8 points on each buccal mucosa, 3 points on the labial mucosa (labial commissures), 6 points on the hard palate, 6 points on the soft palate, and 6 points on the parotid and submandibular glands. PBM treatment was carried out for 21 days, including seven sessions of irradiation until the lesions were healed and all the symptoms disappeared. The VAS scale was assessed before each treatment and 24 h after.

**Figure 1:**
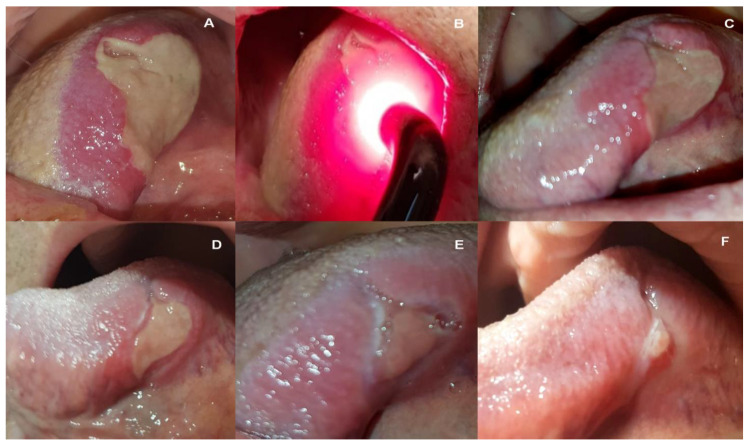
Images of the extensive ulceration on the left lateral surface of the tongue, just before the 1st PBM procedure (**A**), during the 1st PBM (**B**), on the 4th day after the 1st irradiation, and before the 2nd PBM (**C**), on the 7th day before the 3rd PBM treatment (**D**), on the 14th day after the first treatment before the 5th PBM (**E**), and on the 21st day before the 7th and last PBM treatment (**F**).

**Figure 2:**
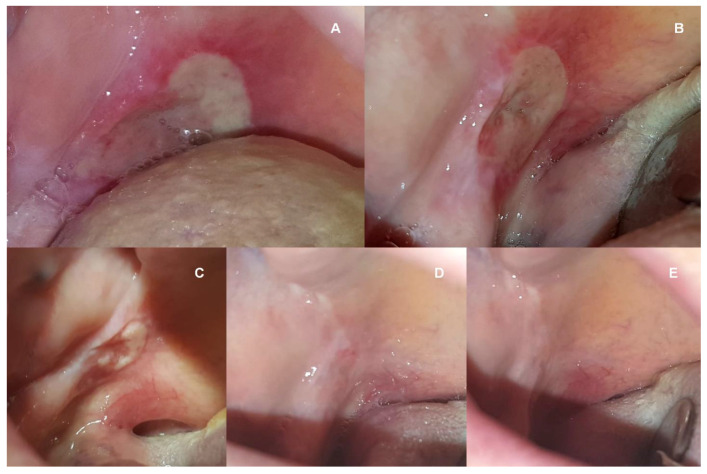
Views of the ulcer on the right pharyngeal arch just before the 1st PBM procedure (**A**), on the 4th day before the 2nd PBM procedure (**B**), on the 7th day before the 3rd PBM (**C**), on the 14th day before the 4th PBM (**D**), and on the 21st before the 7th and last PBM treatment (**E**).

**Figure 3:**
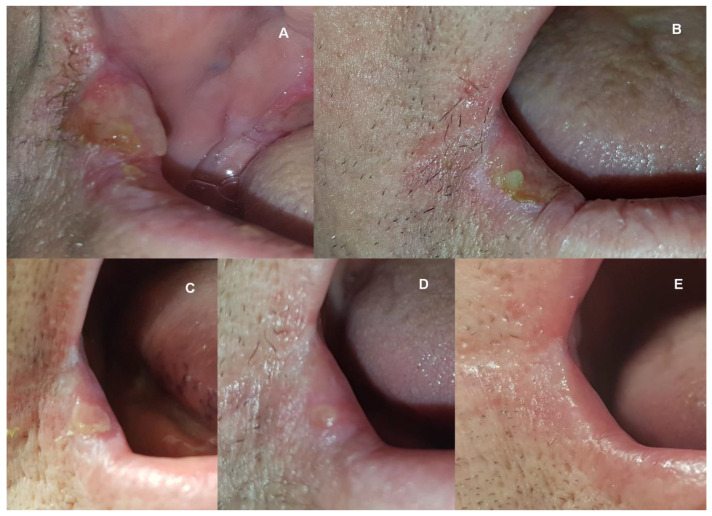
Views of the ulcer in the right labial commissure just before the 1st PBM treatment (**A**), on the 4th day before the 2nd PBM (**B**), on the 7th day before the 3rd PBM (**C**), on the 14th day before the 5th PBM (**D**), and on the 21st day before the 7th and last PBM treatment (**E**).

A systematic diminishing of lesions was observed. On the second PBM visit (4th day), there was a reduction in size to 35 × 28 mm on the tongue, and revascularization was observed under the layer of thick fibrin. On the third PBM visit (7th day), the lesion was about 10 × 6 mm in size; it was surrounded by slight erythema, but no fibrin. Subsequent visits brought further improvements until healing on the 21st day (Figure 1). The situation evolved similarly for a lesion on the soft palate—the 3rd PBM visit resulted in a reduction in size to approx. 15 × 8 mm, with a visible reduction of fibrin and erythema. Already on the 14th day, the mucous membrane showed a preserved continuity with a little redness (Figure 2). In the right labial commissure, on the 4th day, the ulcer decreased to approx. (10 × 6 mm), with trace amounts of fibrin and a narrow inflammation area. On the 7th and 14th days, only slight ulceration was observed. On the 21st day, the lesion had healed, and the mucosa turned pink (Figure 3). The patient reported a significant improvement in comfort caused by the rapid relief of pain, consecutively one day after the 4th day (VAS-4), 7th day (VAS-2), 14th, and 21st day (VAS-0). Oral dryness also disappeared gradually. A saliva tube test was performed in the morning before the patient’s first meal at home; resting saliva was collected for 10 min without stimulation, and stimulated saliva after stimulation by chewing paraffin for 5 min. The quantity of resting and stimulated saliva on the 7th day were 0.25 and 0.5 mL/min, 14th day (0.3 and 0.7 mL/min), and 21st day (0.4 and 1.0 mL/min). The dynamism of healing made it possible to reuse dentures and return to a solid diet. Problems with speech and taste have subsided.

## 3. Discussion

The advancement of OM and related ailments caused a decrease in this patient’s quality of life (QoL). Additionally, the lack of local pharmacological treatment did not improve the patient’s condition. The applied laser settings and PBM regimen initiated immediate and rapid healing despite the small number of visits per week. As in the results of our laboratory tests on human gingival fibroblasts [21], already the third PBM procedure on day 7 resulted in a marked improvement, in the form of shrinkage of all ulcers present on the oral mucosa. An improvement in the form of a significant reduction in pain was noted by the patient on day 4 from the beginning of PBM. Subsequent PBM procedures maintained the simultaneous pace of ulcer healing, pain remission, and increased saliva production.

In the available literature, in the case of severe symptoms, two sessions of PBM per week are a good algorithm, but more often, three appointments per week or daily PBM therapy for the first five days and then every other day is recommended [4,18]. Due to the great distance and general health, the patient’s family did not agree to more frequent visits. More frequent appointments support better cellular and tissue stimulation at a constant level (anti-inflammatory, analgesic, and regenerative effect), although there is a need to be careful and not exceed the metabolic inhibition threshold [2].

According to the literature, red light penetrates all mucosa layers deep enough to reach the tissues effectively by intraoral and extraoral access [2,14,22,23]. Other alternative lengths are near-infrared (NIR); the exact efficacy of the red light (630–670 nm) and NIR (780–830 nm) has been demonstrated, although the optimal energy doses used (at that time) should be different [4].

The parameters of the red laser settings in this patient’s therapy corresponded to the range of doses recommended in the protocol that was suggested by a multinational panel of experts in the field of photobiomodulation and supportive care in cancer patients. The proposed protocol: laser power of 10–150 mW, the energy density of 2–4 J/cm^2^ (no more than 6 J/cm^2^), and exposure time of 20–60 s per point [4]. The presented protocol, recommended by experts, has such a wide range of settings (e.g., output power, energy density) that it is quite difficult to choose individual parameters appropriately tailored to individual patients. Therefore, the applied regimen treatment was based on the results of our laboratory experiments, which allowed us to establish the most optimal stimulation of fibroblasts. The research concerned many variables (wavelength, fluence, output power, irradiation time, number, and frequency of used repetitions). Having selected the protocol in the laboratory, we wanted to confirm its efficacy in vivo. Mobadder et al. [19], in case series reports of advanced OM, also used this (the suggested PBM) protocol with good treatment effect—fast and uncomplicated healing of oral lesions. Emission type low-frequency pulsed light (<100 Hz) has shown a slight advantage over CW light in the prevention of injury and wound healing rate [4], but unfortunately, the laser we used was not able to provide (set) pulse mode but only CW or gated mode.

Although in our case the laser was applied only for advanced lesions, studies also show the possibility of preventive use of the laser before and during CT and RT conducted on the entire mucous membrane, which significantly reduces the risk of advanced OM and reduces its duration [23,24,25]. Martins et al. analyzed the cost-effectiveness of OM treatment of a cycle of PBM treatments compared to pharmacological therapy based on a mouthwash with 0.12% chlorhexidine and oral ointment with hydrocortisone 5 mg, neomycin 5 mg, troxerutin 20 mg, ascorbic acid 0.5 mg, and benzocaine 2 mg. PBM therapy is not only a more effective treatment, but is also advantageous in terms of costs which are much lower for a single oncological patient and the entire health care system [22,26].

The scientific evidence for the anti-inflammatory, analgesic, regenerative effects, and stimulation locally the patient’s immune system of lasers is unequivocal enough that the Clinical Practice Guidelines of the (MASCC/ISOO). Mucositis Study Group recommends PBM for the prevention and treatment of OM in HSCT recipients conditioned with high-dose chemotherapy, with or without total body irradiation, and suggests a positive role for patients treated with RT for head and neck cancer [5,6]. Easy access to the inside of the mouth, painless, repeatability, and no side effects, are the strengths of PBM. Some limitations are caused by the necessity to open the mouth for a long time with a large extent of symptoms, limited access to the areas near the throat and behind the tongue, and the necessity to perform a long therapeutic cycle.

## 4. Conclusions

The results presented here confirm the efficiency of the protocol and laser settings tested. Our case shows that the use of PMB therapy contributes to faster healing of painful oral lesions in oncological patients, and thus their treatment time and return to the appropriate QoL is shorter. Further studies need to be done on more severe oncological patients to identify the optimal parameters and treatment protocol unequivocally [27].

## Abbreviations

OM—oral mucositis, PBM—photobiomodulation, CT—chemotherapy, RT—radiotherapy, HSCT—hematopoietic stem cell transplantation, WHO—World Health Organization, NSI-CTCAE—National Cancer Institute-Common Terminology Criteria for Adverse Events, DLBCL—diffuse large B-cell lymphoma, R-IPI—revised international prognostic index, PET—Positron Emission Tomography, VAS—visual analog scale, MASCC/ISOO/ESMO—Multinational Association of Supportive Care in Cancer/International Society of Oral Oncology/European Society for Medical Oncology, ASCO—American Society of Clinical Oncology, QoL—patient’s quality of life.

## References

[1] Sonis S.T., Elting L.S., Keefe D., Peterson D.E., Schubert M., Hauer-Jensen M., Bekele B.N., Raber-Durlacher J., Donnelly J.P., Rubenstein E.B. (2004). Mucositis Study Section of the Multinational Association for Supportive Care in Cancer; International Society for Oral Oncology. Perspectives on cancer therapy-induced mucosal injury: Pathogenesis, measurement, epidemiology, and consequences for patients. Cancer.

[2] Zecha J.A., Raber-Durlacher J.E., Nair R.G., Epstein J.B., Sonis S.T., Elad S., Hamblin M.R., Barasch A., Migliorati C.A., Milstein D.M. (2016). Low-level laser therapy/photobiomodulation in the management of side effects of chemoradiation therapy in head and neck cancer: Part 1: Mechanisms of action, dosimetric, and safety considerations. Support Care Cancer.

[3] Melton C. (2017). Chemotherapy-Induced and Radiotherapy-Induced Oral Mucositis. https://www.cancertherapyadvisor.com/home/cancer-topics/supportive-care/side-effect-management/chemotherapy-induced-and-radiotherapy-induced-oral-mucositis/4/.

[4] Zecha J.A., Raber-Durlacher J.E., Nair R.G., Epstein J.B., Elad S., Hamblin M.R., Barasch A., Migliorati C.A., Milstein D.M., Genot M.T. (2016). Low-level laser therapy/photobiomodulation in the management of side effects of chemoradiation therapy in head and neck cancer: Part 2: Proposed applications and treatment protocols. Support Care Cancer.

[5] Lalla R.V., Bowen J., Barasch A., Elting L., Epstein J., Keefe D.M., McGuire D.B., Migliorati C., Nicolatou-Galitis O., Peterson D.E. (2014). MASCC/ISOO clinical practice guidelines for the management of mucositis secondary to cancer therapy. Cancer.

[6] Elad S., Cheng K.K.F., Lalla R.V., Yarom N., Hong C., Logan R.M., Bowen J., Gibson R., Saunders D.P., Zadik Y. (2020). MASCC/ISOO clinical practice guidelines for the management of mucositis secondary to cancer therapy. Cancer.

[7] Correa M.E.P., Cheng K.K.F., Chiang K., Kandwal A., Loprinzi C.L., Mori T., Potting C., Rouleau T., Toro J.J., Ranna V. (2020). Systematic review of oral cryotherapy for the management of oral mucositis in cancer patients and clinical practice guidelines. Support Care Cancer.

[8] Peterson D.E., Ohrn K., Bowen J., Fliedner M., Lees J., Loprinzi C., Mori T., Osaguona A., Weikel D.S., Elad S. (2013). Mucositis Study Group of the Multinational Association of Supportive Care in Cancer/International Society of Oral Oncology (MASCC/ISOO). A systematic review of oral cryotherapy for management of oral mucositis caused by cancer therapy. Support Care Cancer.

[9] Grzech-Leśniak K., Bencharit S., Dalal N., Mroczka K., Deeb J.G. (2019). In-Vitro Examination of the Use of Er: YAG Laser to Retrieve Lithium Disilicate Crowns from Titanium Implant Abutments. J. Prosthodont..

[10] Golob Deeb J., Smith J., Belvin B.R., Lewis J., Grzech-Leśniak K. (2019). Er: YAG Laser Irradiation Reduces Microbial Viability When Used in Combination with Irrigation with Sodium Hypochlorite, Chlorhexidine, and Hydrogen Peroxide. Microorganisms.

[11] Deeb J.G., Bencharit S., Dalal N., Abdulmajeed A., Grzech-Leśniak K. (2019). Using Er: YAG laser to remove lithium disilicate crowns from zirconia implant abutments: An in vitro study. PLoS ONE.

[12] Deeb J.G., Grzech-Leśniak K., Weaver C., Matys J., Bencharit S. (2019). Retrieval of Glass Fiber Post Using Er: YAG Laser and Conventional Endodontic Ultrasonic Method: An In Vitro Study. J. Prosthodont..

[13] Grzech-Leśniak K., Bencharit S., Skrjanc L., Kanduti D., Matys J., Deeb J.G. (2020). Utilization of Er: YAG Laser in Retrieving and Reusing of Lithium Disilicate and Zirconia Monolithic Crowns in Natural Teeth: An In Vitro Study. Appl. Sci..

[14] Arnabat-Dominguez J., Vecchio A.D., Todea C., Grzech-Leśniak K., Vescovi P., Romeo U., Nammour S. (2021). Laser dentistry in daily practice during the COVID-19 pandemic: Benefits, risks, and recommendations for safe treatments. Adv. Clin. Exp. Med..

[15] Marín-Conde F., Castellanos-Cosano L., Pachón-Ibañez J., Serrera-Figallo M.A., Gutiérrez-Pérez J.L., Torres-Lagares D. (2019). Photobiomodulation with low-level laser therapy reduces oral mucositis caused by head and neck radio-chemotherapy: Prospective randomized controlled trial. Int. J. Oral Maxillofac. Surg..

[16] Bensadoun R.J. (2018). Photobiomodulation or low-level laser therapy in the management of cancer therapy-induced mucositis, dermatitis, and lymphedema. Curr. Opin. Oncol..

[17] de Lima V.H.S., de Oliveira-Neto O.B., da Hora Sales P.H., da Silva Torres T., de Lima F.J.C. (2020). Effectiveness of low-level laser therapy for oral mucositis prevention in patients undergoing chemoradiotherapy for the treatment of head and neck cancer: A systematic review and meta-analysis. Oral. Oncol..

[18] Gobbo M., Verzegnassi F., Ronfani L., Zanon D., Melchionda F., Bagattoni S., Majorana A., Bardellini E., Mura R., Piras A. (2018). A multicenter randomized, double-blind controlled trial to evaluate the efficacy of laser therapy for the treatment of severe oral mucositis induced by chemotherapy in children: LaMPO RCT. Pediatr. Blood Cancer.

[19] Mobadder M.E., Farhat F., El Mobadder W., Nammour S. (2019). Photobiomodulation Therapy in the Treatment of Oral Mucositis, Dysphagia, Oral Dryness, Taste Alteration, and Burning Mouth Sensation Due to Cancer Therapy: A Case Series. Int. J. Environ. Res. Public Health.

[20] Dompe C., Moncrieff L., Matys J., Grzech-Leśniak K., Kocherova I., Bryja A., Bruska M., Dominiak M., Mozdziak P., Skiba T.H.I. (2020). Photobiomodulation-Underlying Mechanism and Clinical Applications. J. Clin. Med..

[21] Kocherova I., Bryja A., Błochowiak K., Kaczmarek M., Stefańska K., Matys J., Grzech-Leśniak K., Dominiak M., Mozdziak P., Kempisty B. (2021). Photobiomodulation with Red and Near-Infrared Light Improves Viability and Modulates Expression of Mesenchymal and Apoptotic-Related Markers in Human Gingival Fibroblasts. Materials.

[22] Mobadder M.E., Farhat F., Mobadder W.E., Nammour S. (2018). Photobiomodulation Therapy in the Treatment of Oral Mucositis, Dysgeusia and Oral Dryness as Side-Effects of Head and Neck Radiotherapy in a Cancer Patient: A Case Report. Dent. J..

[23] Oberoi S., Zamperlini-Netto G., Beyene J., Treister N.S., Sung L. (2014). Effect of prophylactic low-level laser therapy on oral mucositis: A systematic review and meta-analysis. PLoS ONE.

[24] He M., Zhang B., Shen N., Wu N., Sun J. (2018). A systematic review and meta-analysis of the effect of low-level laser therapy (LLLT) on chemotherapy-induced oral mucositis in pediatric and young patients. Eur. J. Pediatr..

[25] Migliorati C., Hewson I., Lalla R.V., Antunes H.S., Estilo C.L., Hodgson B., Lopes N.N.F., Schubert M.M., Bowen J., Elad S. (2013). Systematic review of laser and other light therapy for the management of oral mucositis in cancer patients. Support Care Cancer.

[26] Martins A.F.L., Nogueira T.E., Morais M.O., Oton-Leite A.F., Valadares M.C., Batista A.C., Freitas N.M.A., Leles C.R., Mendonça E.F. (2019). Effect of photobiomodulation on the severity of oral mucositis and molecular changes in head and neck cancer patients undergoing radiotherapy: A study protocol for a cost-effectiveness randomized clinical trial. Trials.

[27] Patini R., Staderini E., Camodeca A., Guglielmi F., Gallenzi P. (2019). Case Reports in Pediatric Dentistry Journals: A Systematic Review about Their Effect on Impact Factor and Future Investigations. Dent. J..

